# Low dose slow and ultraslow thrombolytic therapy for patients with prosthetic valve thrombosis: a pharmacist-led protocol experience from a Middle Eastern quaternary care center

**DOI:** 10.3389/fcvm.2026.1732701

**Published:** 2026-03-18

**Authors:** Ziad Sadik, Khaled Al Zaman, Emna Abidi, Mohammed Khalil, Firas Al Badarin, Praveen Ghisulal, Bassam Atallah

**Affiliations:** 1 Department of Pharmacy Services, Cleveland Clinic Abu Dhabi, Abu Dhabi, United Arab Emirates; 2 Internal Medicine Department, Sheikh Shakhbout Medical City, Abu Dhabi, United Arab Emirates; 3 Cleveland Clinic Lerner College of Medicine of Case, Western Reserve University, Cleveland, OH, United States; 4 Heart, Vascular & Thoracic Institute, Cleveland Clinic Abu Dhabi, Abu Dhabi, United Arab Emirates; 5 Critical Care Institute, Cleveland Clinic Abu Dhabi, Abu Dhabi, United Arab Emirates; 6 Clinical Affairs, College of Pharmacy, Dubai Medical University, Dubai, United Arab Emirates

**Keywords:** alteplase, efficacy, prosthetic valve, thrombolytic therapy, thrombosis

## Abstract

**Background:**

Slow and ultraslow infusions of low-dose thrombolytic therapy (TT) have emerged as promising alternatives for managing mechanical prosthetic valve thrombosis (PVT), potentially enhancing thrombus resolution while reducing bleeding complications. This study presents real-world clinical outcomes using low-dose alteplase regimens administered according to a pharmacist-led institutional protocol.

**Methods:**

A retrospective cross-sectional study was conducted at a multispecialty quaternary care hospital in the United Arab Emirates. Twelve patients presenting with heart failure symptoms and confirmed PVT received low-dose alteplase thrombolytic regimens. The protocol involved either a slow infusion (25 mg over 6 hours) or an ultraslow infusion (25 mg over 25 hours). Clinical characteristics, treatment regimens, and outcomes were assessed.

**Results:**

Most patients presented with New York Heart Association (NYHA) class II and III symptoms, representing 50% and 41.7% of cases, respectively. Mitral valve thrombosis was observed in 58.3% of patients, aortic valve thrombosis in 33.3%, and one patient exhibited thrombi involving both valves. The ultraslow infusion protocol was applied in 91.7% of cases. The median total alteplase dose administered was 47.9 mg (range 25 to 125 mg), delivered over one to five doses. Clinical success, defined as complete or partial resolution of valve thrombosis, was achieved in 50% of patients after the first dose and 83.3% after the second dose. One patient experienced an ischemic stroke. Bleeding complications occurred in one third of patients, with only one major bleeding event. All patients survived at one-month follow-up, and one patient required valve surgery.

**Conclusion:**

Low-dose slow and ultraslow alteplase thrombolytic regimens demonstrated favorable clinical outcomes in patients with symptomatic PVT within a real-world clinical setting. Despite limitations inherent to a retrospective observational design and small sample size, these findings support pharmacologic thrombolysis as a potential alternative to surgery in selected patients. Larger prospective studies are required to further define optimal thrombolytic regimens and patient selection criteria.

## Background

Prosthetic valve thrombosis (PVT) remains a major challenge after mechanical heart valve implantation despite demonstrable superior durability compared with bioprosthetic valves. Systemic anticoagulation remains the mainstay of PVT prevention and the occurrence of PVT continues to pose significant morbidity and mortality, including stroke, heart failure, and death. The overall prevalence of PVT reaches 5.7% (1). Meanwhile, the prevalence of valve thrombosis after surgical bioprosthetic valve replacement reaches up to 0.61–0.7% (1, 2).

While reoperation has historically been the only available treatment option for symptomatic and unstable patients with PVT, recent advancements in low-dose thrombolytic therapy (TT), with agents like alteplase, offer promising alternatives. Slow and ultraslow alteplase infusion regimens have shown efficacy in resolving PVT while minimizing risk of hemorrhagic complications. Furthermore, in the event of symptomatic left-sided PVT, the 2020 ACC/AHA guidelines recommend urgent initial treatment with either slow infusion, low-dose thrombolytic therapy, or emergency surgery, while the most recent European Society of Cardiology/EACTS guidelines still prioritize surgery in the management of PVT (2–6). Ultraslow protocols were proposed as the means to decrease complication rates without affecting success rates in a subset of patients requiring lytic therapy (2, 3).

Despite the high prevalence of rheumatic heart disease and use of mechanical valve prostheses in the Middle East and Gulf region compared to North America and Europe, data on the utilization and outcomes of various thrombolytic protocols for patients with prosthetic valve thrombosis (PVT) remain limited. Over the past decade, evidence from the TROIA trial (2013), the PROMETEE study (2015), and the HATTUSHA study (2022) has demonstrated the safety and efficacy of tailored low-dose slow and ultraslow alteplase infusion strategies for PVT management (7–9). These studies have shaped contemporary clinical practice by emphasizing individualized infusion duration based on patient stability and thrombus characteristics rather than fixed thrombolytic dosing. The institutional thrombolytic strategy and decision-making algorithm were developed using the practical frameworks described in these studies. The present report describes the safety and feasibility of an institutional protocol for treating PVT utilizing 2 different alteplase infusion protocols.

## Methods

### Study design and population

**Figure 1:**
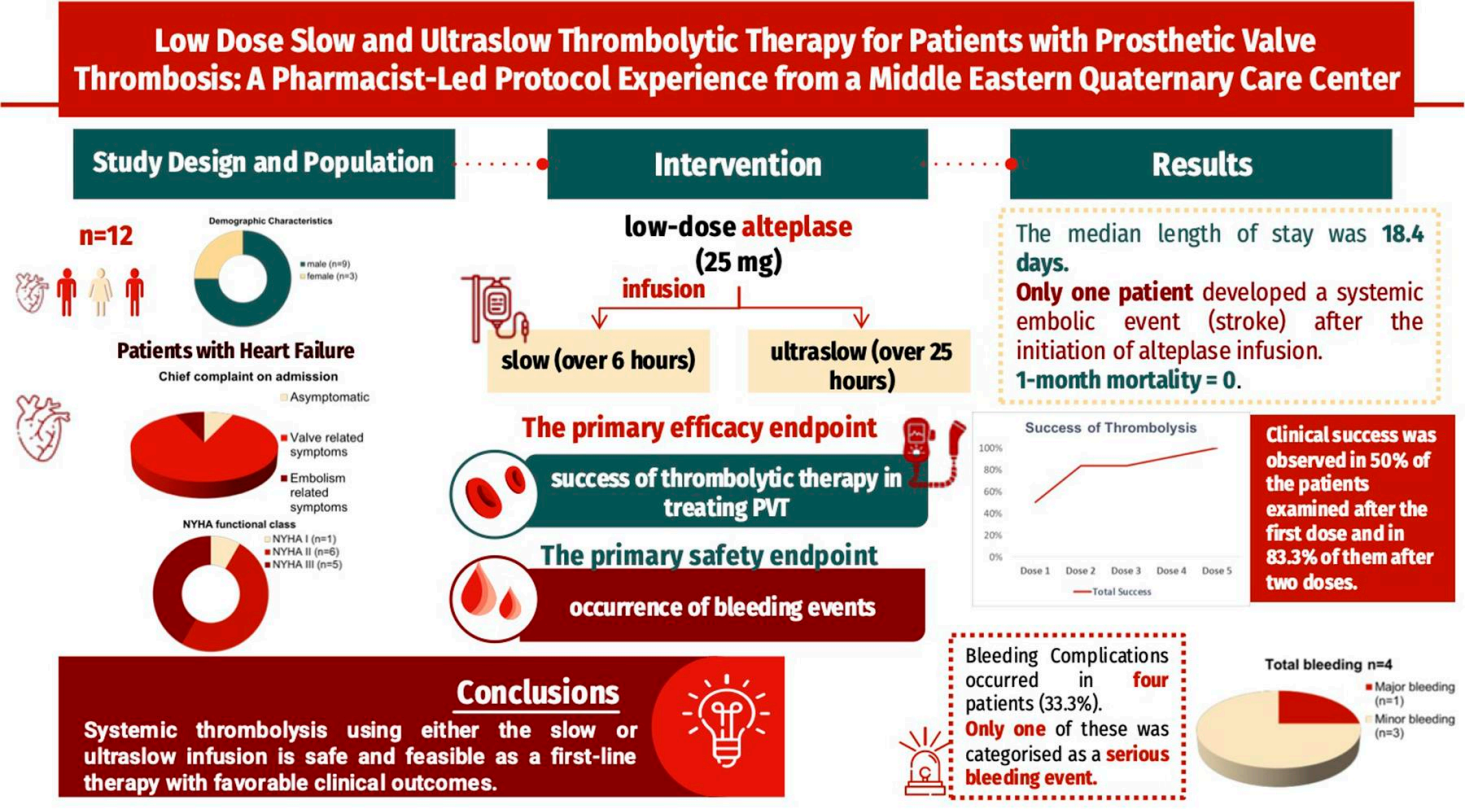
Investigating the efficacy of low-dose thrombolytic therapy (TT) in prosthetic valve thrombosis (PVT): this study examines the real-world outcomes of a slow (6 h) and an ultra-slow (25 h) infusion of alteplase (25 mg) in 12 patients with heart failure symptoms. Key findings included a clinical success rate of 50% after one dose and 83.3% after two doses. Bleeding complications occurred in four patients, with only one major bleeding event. Remarkably, all patients survived after one month of follow-up, emphasizing the potential of tailored thrombolytic therapies in the treatment of PVT.

This was a single-center, retrospective study conducted at a multispecialty quaternary care hospital (Abu Dhabi, UAE). We retrospectively identified eligible adult patients who received low-dose alteplase (defined as 25 mg) administered as either a slow infusion (over 6 h) or ultraslow infusion (over 25 h) infusion for the indication of prosthetic valve thrombosis between July 2018 and June 2024. Exclusion criteria included patients with incomplete data sets, those prescribed alteplase for indications other than PVT, and those with coagulation abnormalities such as collagen disease or protein C deficiency. The study was approved by the Institutional Review Board and Research Ethics Committee, with waiver of informed consent due to the retrospective nature of the study (Figure 1).

### Data collection

Patients were identified through a structured retrospective search of the institutional electronic medical record system (EPIC). Patient identification and data extraction were performed directly from this system and were not based on a predefined external registry or dataset.

The collected variables included baseline demographics, prosthetic valve type and position, elapsed time since valve surgery, cardiac rhythm status, the alteplase regimen administered, concomitant medications, New York Heart Association (NYHA) functional class at presentation, history of thrombosis and bleeding, relevant laboratory parameters, transesophageal echocardiography data, and in hospital adverse events.

### Diagnostic criteria for prosthetic valve thrombosis

Diagnosis of prosthetic valve thrombosis was established through a multimodality imaging approach prior to initiation of thrombolytic therapy. Clinical suspicion was based on the presence of dyspnea, heart failure symptoms, embolic events, new prosthetic valve murmurs, or subtherapeutic anticoagulation.

All patients underwent transthoracic echocardiography, which demonstrated elevated transvalvular gradients compared to baseline and evidence of impaired prosthetic valve hemodynamics. Definitive confirmation was obtained with transesophageal echocardiography, enabling direct visualization of thrombus, measurement of thrombus size and mobility, and differentiation from pannus formation or other structural abnormalities.

The study included only patients who demonstrated echocardiographic evidence of thrombus in addition to either obstructive or non-obstructive prosthetic valve dysfunction.

### Thrombolytic therapy and concomitant anticoagulation

The selection between low dose slow and ultraslow alteplase infusion was determined by a predefined internal clinical policy, developed based on the available evidence at the time, initially drawing on experience from TROIA (2013) and PROMETEE (2015), and subsequently informed by the HATTUSHA study (2022), which evaluated the safety and outcomes of tailored low-dose infusions in prosthetic valve thrombosis (PVT).This evidence base informed both the clinical selection between slow and ultraslow protocols and the timing of their integration into the computerized protocol. This decision pathway incorporated clinical parameters such as NYHA functional class, hemodynamic stability, thrombus burden and mobility as assessed by transesophageal echocardiography, and the presence of obstructive prosthetic valve physiology. The complete institutional protocol is provided in the Supplementary Material.

Alteplase was administered at a dose of 25 mg per infusion using either a slow infusion protocol over 6 h or an ultraslow infusion protocol over 25 h. Patients with NYHA class I or II symptoms were preferentially treated with the ultraslow infusion protocol, whereas those with class III or IV symptoms received the slow infusion protocol. Repeat alteplase infusions were administered in cases of partial or absent clinical or echocardiographic response, at the discretion of the treating team. Although institutional practice allowed for administration of up to eight doses corresponding to a cumulative dose of 200 mg, the maximum number of doses administered in this study cohort was five.

Transesophageal echocardiography was performed in all patients prior to initiation of thrombolytic therapy for confirmation of prosthetic valve thrombosis and assessment of valve hemodynamics. Follow up echocardiographic assessment was performed after each alteplase infusion to guide subsequent treatment decisions. Intravenous unfractionated heparin was initiated following completion of each alteplase infusion.

All oral anticoagulants, including warfarin, were withheld prior to initiation of the alteplase infusion in accordance with institutional protocol. No oral anticoagulant therapy was administered during thrombolytic treatment. Anticoagulation management following completion of thrombolysis was individualized based on clinical status, bleeding risk, and echocardiographic response.

### Outcomes

Outcomes were evaluated using predefined clinical and echocardiographic criteria applied to existing medical records. Clinical success of thrombolytic therapy was defined as the presence of symptomatic improvement together with objective echocardiographic evidence of improved prosthetic valve hemodynamics, including a reduction in transvalvular gradients. Complete response was defined as normalization or near normalization of transvalvular gradients with restoration of normal leaflet motion and absence of visible thrombus on follow up imaging. Partial response was defined as a significant improvement in transvalvular gradients and or leaflet mobility with residual thrombus or persistent, though improved, hemodynamic abnormalities. Safety outcomes focused on the occurrence of bleeding events during or following thrombolytic therapy.

Secondary outcomes included length of hospital stay, systemic embolic complications, need for valve reoperation, and all cause mortality at one month. Major bleeding events were classified according to the International Society on Thrombosis and Haemostasis (ISTH) criteria.

### Statistical analysis

Descriptive statistics were used to summarize demographic, clinical, and echocardiographic variables. Continuous variables were reported as mean with standard deviation or median with range or interquartile range, as appropriate. Categorical variables were summarized using frequencies and percentages. Given the descriptive nature and small sample size of the study, no inferential statistical testing was performed.

## Results

A total of 12 patients were identified (8/12 men; 66.7%) with an average age of 47.8 ± SD years. Most patients presented with either NYHA class II or III symptoms, 50% and 41.7%, respectively. Only one patient had a previous stroke prior to alteplase infusion.

Seven patients had prosthetic valve thrombosis involving the mitral valve prosthesis, four involved the aortic valve prosthesis, and one patient had thrombosis involving both valves. Among the aortic valve prostheses, four were St Jude mechanical valves and one was an On X mechanical valve. Of the mitral valve prostheses, six were St Jude mechanical valves and two were On X mechanical valves. The median elapsed time since valve surgery was 44.2 months (range 1 to 192 months). At presentation, ten patients had valve related symptoms, primarily reflecting heart failure manifestations such as dyspnea, pulmonary congestion, and peripheral edema, one patient was asymptomatic, and one patient presented with embolic symptoms in the form of a middle cerebral artery cardioembolic ischemic stroke. All diagnoses of prosthetic valve thrombosis were made during hospital admission as part of symptom driven evaluation. The median baseline INR at the time of admission was 2.3 (IQR 1.2 to 7.5), which was subsequently reduced to 1.4 (IQR 1.1 to 2.4) prior to initiation of thrombolytic therapy (Table 1).

**Table 1:** Baseline characteristics of the studied patients.

Demographic characteristics	*n* = 12
Age	47.8 (30–66)
Gender	
Male	9 (75.0%)
Female	3 (25.0%)
BMI, kg/m^2^	25.1 (21.7–29.7)
ETSVS, months	44.2 (1–192)
NYHA functional class	
I	1 (8.3%)
II	6 (50.0%)
III	5 (41.7%)
Chief complaint on admission	
Asymptomatic	1 (8.3%)
Valve related symptoms	10 (83.4%)
Embolism related symptoms	1 (8.3%)
History of stroke	
Before infusion	1 (8.3%)
After infusion	1 (8.3%)
No history of stroke	10 (83.4%)
Comorbidities	
DM	3 (25.0%)
CAD	2 (16.7%)
HTN	4 (33.3%)
Dyslipidemia	5 (41.7%)
AFib	7 (58.3%)
CHF	8 (66.7%)
Thrombosed valve location	
Mitral Valve Replacement	7 (58.3%)
Aortic Valve Replacement	4 (33.3%)
Mitral and Aortic Valve Replacement	1 (8.3%)
Labs	
Admission INR, Aortic Valve	2.2 (1.3–3.6)
Admission INR, Mitral Valve	2.2 (1.2–7.5)
INR on day of infusion, Aortic valve	1.6 (1.2–2.4)
INR on day of infusion, Mitral valve	1.4 (1.2–1.5)
White blood cell count, 10^9^/L	11.5 (4.7–26.2)
Platelets, 10^9^/L	212.3 (121–351)
APPT (seconds)	59.1 (26.4–180)
CRP, mg/L	100.9 (3.42–349.5)
Glucose, mmol/L	7.5 (4.3–16.9)
Creatinine, µmol/L	100.9 (56–203)
AST, U/L	29.5 (15–58)
ALT, U/L	23.8 (8–62)

BMI, body mass index; ETVS, elapsed time since valve replacement; DM, diabetes mellitus; CAD, coronary artery disease; HTN, hypertension; AFib, atrial fibrillation; CHF, congestive heart failure; INR, international normalized ratio; APPT, activated partial thromboplastin clotting time; CRP, C-reactive protein; AST, aspartate aminotransferase; ALT, alanine transaminase.

### Alteplase protocol and success rate

Ten patients received the ultra-slow infusion protocol, 1 patient the slow infusion protocol, and 1 patient was switched from the ultra-slow to slow infusion protocol after an inadequate response to the second dose. The median cumulative alteplase dose administered was 47.9 mg (IQR 25 to 125 mg), corresponding to a total of 1 to 5 doses per patient (Table 2).

**Table 2:** Alteplase regimen.

Alteplase regimen characteristics	Results
Slow protocol	2 (16.7%)
Ultraslow protocol	11 (91.7%)
Slow protocol dose (mg)	50 (25–75)
Ultraslow protocol dose (mg)	43.2 (25–100)
Total alteplase dose (mg)	47.9 (25–125)

**Figure 2:**
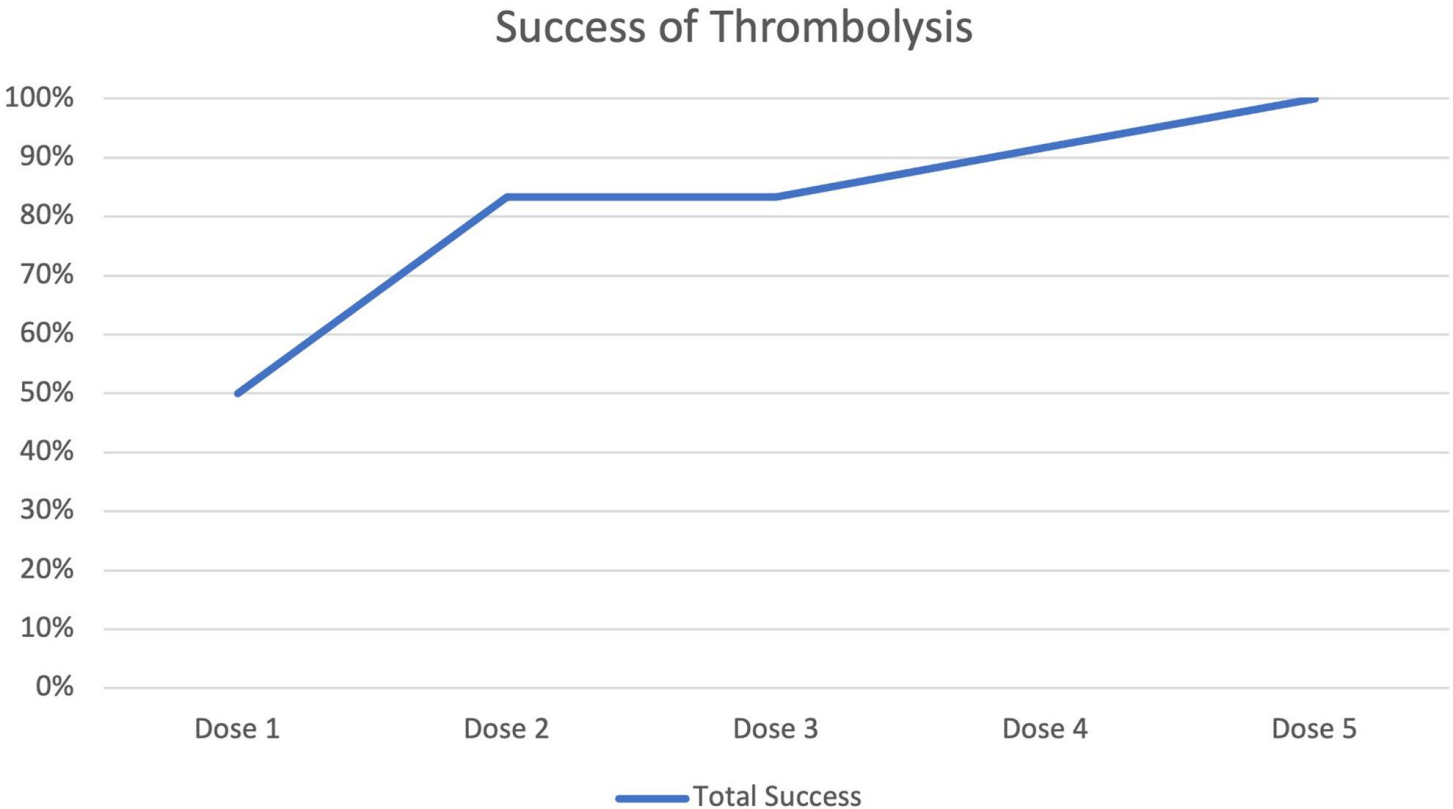
Success of thrombolysis.

Clinical success was noted in 50% of the studied patients after the first dose and in 83.3% of them after two doses. The remaining two patients required 4 and 5 doses, respectively (Table 3 and Figure 2).

**Table 3:** Success of alteplase.

Treatment outcome	Slow (*n* = 1)	Ultraslow (*n* = 10)	Switch (*n* = 1)	Total
Success after 1st dose	1	5	0	6 (50%)
Additional success after 2nd dose	0	4	0	10 (83.3%)
Additional success after 4th dose	0	1	0	11 (91.7%)
Additional success after 5th dose	0	0	1	12 (100%)
Total success	1	10	1	12 (100%)

### Clinical results

The median length of hospital stay was 18.4 days (7–38 days). One patient experienced a systemic embolic complication (ischemic stroke) following initiation of alteplase therapy.‏ No cases of haemorrhagic stroke or haemorrhagic transformation were observed in our cohort. Bleeding complications occurred in four patients (33.3%), including hemoptysis in two patients, epistaxis in one patient, and rectal bleeding in one patient. Of these events, only one patient who had multiple episodes of epistaxis met criteria for major bleeding, as it required multiple blood transfusions. One patient who underwent mechanical aortic valve replacement ultimately required surgical valve intervention (Table 4).

**Table 4:** Safety outcomes.

Outcome	Value
Hospital stay, days, median (range)	18.4 (7–38)
Systemic embolic complications, *n* (%)	1 (8.3%)
Total bleeding, *n* (%)	4 (33.3%)
Major bleeding, *n* (%)	1 (8.3%)
Minor bleeding, *n* (%)	3 (25%)
Required valve surgical intervention, *n* (%)	1 (8.3%)
1-month mortality, *n* (%)	0 (0%)

Serial transesophageal echocardiographic assessment demonstrated improvement in prosthetic valve hemodynamics following thrombolytic therapy. The mitral valve maximum gradient showed minimal change, decreasing from an initial value of 32.9 mmHg to 32.6 mmHg at follow up. However, the mitral valve mean gradient markedly decreased from 22.3 mmHg to 5.6 mmHg. More pronounced improvement was observed in aortic valve gradients, with the maximum gradient decreasing from 62.8 mmHg to 9.6 mmHg and the mean gradient decreasing from 36.4 mmHg to 20.6 mmHg. Left ventricular ejection fraction remained stable throughout treatment, with mean values of 49.4% at baseline and 49.3% at follow up. A summary of echocardiographic findings is provided in (Table 5).

**Table 5:** Transesophageal echocardiography (TEE) data.

Echocardiographic parameter	Value
Mitral valve initial max gradient, mmHg (range)	32.9 (13–54)
Mitral valve final max gradient, mmHg (range)	32.6 (13–55)
Mitral valve initial mean gradient, mmHg (range)	22.3 (10–39)
Mitral valve final mean gradient, mmHg (range)	5.6 (2–10)
Aortic valve initial max gradient, mmHg (range)	62.8 (21–123)
Aortic valve final max gradient, mmHg (range)	9.6 (4–16)
Aortic valve initial mean gradient, mmHg (range)	36.4 (14–69)
Aortic valve final mean gradient, mmHg (range)	20.6 (9–36)
Initial LV ejection fraction %	49.4 (12–66)
Final LV ejection fraction %	49.3 (14–70)
Initial RV function	
Normal	8 (66.7%)
Mildly reduced	1 (8.3%)
Moderately reduced	2 (16.7%)
Moderately to severely reduced	1 (8.3%)
Final RV function	
Normal	6 (50.0%)
Mildly reduced	4 (33.4%)
Moderately reduced	1 (8.3%)
Not documented	1 (8.3%)

Right ventricular function was assessed using standard echocardiographic parameters in accordance with current echocardiography guidelines. Parameters included tricuspid annular plane systolic excursion (TAPSE), right ventricular fractional area change (FAC), and visual qualitative assessment of right ventricular systolic function. Right ventricular dysfunction was defined as reduced TAPSE, reduced FAC, or qualitative evidence of impaired systolic function.

Regarding RV function, initially, 66.7% of patients had normal RV function, but by the end, only 50% remained in the normal range, while the proportion of those with mildly reduced function increased from 8.3% to 33.3%. Moderate reductions in RV function were relatively unchanged.

## Discussion

The current study describes experience with hospital wide implementation of a TT protocol, that provides clear guidance to clinicians on dosing instruction and necessary laboratory studies prior to initiation of TT for PVT. Furthermore, it demonstrates the safety and efficacy of this protocol in treating hemodynamically stable patients with PVT, and shows that up to 83% of patients with PVT experience significant improvement in prosthetic valve hemodynamics after the second TT infusion. Overall, this is achieved without the occurrence of significant bleeding complications. As such, our data provide much-needed assurance that repeating TT infusions, up to five rounds per our study, was feasible and safe and did not significantly increase bleeding complications.

Valve thrombosis arises from adsorbing plasma proteins like fibrinogen, fibronectin, and von Willebrand factor onto the valve surface, triggering a cascade of platelet activation and coagulation (5). While long-term use of systemic anticoagulation significantly decreases the risk of PVT, poor adherence to oral anticoagulation is a significant risk factor for PVT.

Although direct comparison of TT with surgery presents challenges, studies have demonstrated promising outcomes with TT. However, controversy persists regarding the optimal type, dose, rate, and route of administration (7, 10–12). Currently, no universally accepted TT strategy exists, and reliable predictors of bleeding and other complications remain unidentified. Furthermore, precise clinical and echocardiographic predictors of TT complications, including thromboembolism and bleeding, have not been clearly established.

Several studies have evaluated the safety and efficacy of TT regimens for treating PVT. The TROIA trial (Comparison of Different Transesophageal Echocardiography Guided Thrombolytic Regimens for Prosthetic Valve Thrombosis), the largest PVT trial to date, demonstrated the efficacy and safety of low-dose (25 mg), slow (6-hour) rtPA infusions without bolus, repeated as needed, compared to higher doses or faster infusions (7, 12). Similarly, the PROMETEE trial (A novel strategy in the management of Prosthetic Mechanical valve thrombosis and the predictors of outcome) evaluated the ultraslow (25-h) infusion of low-dose (25 mg) rtPA and identified this regimen as safe and effective for most PVT patients, except those with severe heart failure (8). Notably, the HATTUSHA study, a multicenter, non-randomized observational prospective study that performed slow (6 h) and/or ultraslow (25 h) infusion of tPA (25 mg) TT regimen on obstructive PVT patients in repeated sessions, associated prolonged low-dose TT infusions with high success rates, lower complications, and reduced mortality compared to surgery for obstructive PVT in various functional classes. Moreover, the study results were in accordance with an effective treatment in NYHA functional class I-IV patients with obstructive PVT, in the absence of contraindications, and with the possibility of performing a combination of the 2 regimens (9).

The current study has several limitations. First, the small sample size precludes adequately powered statistical analyses and limits the ability to identify independent predictors of successful thrombolytic therapy or adverse outcomes. Second, the retrospective and single center design may introduce selection bias and limits the generalizability of the findings to other institutions and patient populations. Third, treatment allocation was not randomized and was influenced by clinical judgment and symptom severity, which may have resulted in confounding by indication. In addition, patients perceived to be at higher baseline risk for hemorrhagic complications may have been preferentially directed toward alternative management strategies and therefore not captured in this analysis.

Furthermore, detailed echocardiographic measurements of thrombus size and mobility were not consistently available for all patients due to the retrospective nature of data collection and reliance on clinical documentation, which limits more granular assessment of thrombus characteristics and their relationship to outcomes. Despite these limitations, this study represents, to our knowledge, the largest reported case series evaluating the feasibility and safety of structured thrombolytic therapy protocols for prosthetic valve thrombosis in the Middle East and Gulf region, and provides important real world data that may serve as a foundation for future multicenter and prospective investigations.

## Conclusion

Our findings suggest that systemic thrombolysis using either the slow or ultraslow infusion is safe and feasible as a first-line therapy with favorable clinical outcomes. Future adequately powered studies will further corroborate the benefits of TT in this population and clarify the criteria for the selection of slow vs. ultraslow infusion.

## Data Availability

The raw data supporting the conclusions of this article will be made available by the authors, without undue reservation.
