# Supplementary material for: Low dose slow and ultraslow thrombolytic therapy for patients with prosthetic valve thrombosis: a pharmacist-led protocol experience from a Middle Eastern quaternary care center
Source: Front Cardiovasc Med. 2026 Mar 18;13:1732701. doi: 10.3389/fcvm.2026.1732701 (PMC13038972; doi:10.3389/fcvm.2026.1732701)
Supplement: Supplementary file 1 [file Datasheet1.pdf]

Protocol

- Consideration for use of slow and ultraslow alteplase for prosthetic mechanical valve thrombosis:

  1.1 Obstructive thrombus or non-obstructive thrombus (with or without a history of recent thromboembolism and a thrombus diameter of ≥10 mm).
  1.2 Ultraslow (25 hours) infusion of low-dose (25 mg) t-PA without bolus appears to be associated with quite low nonfatal complications and mortality
  1.3 The success rate of this thrombolytic therapy has been reported as up to 90%
  1.4 NYHA Class IV is a predictor of thrombolytic therapy failure

- Contraindications to alteplase use in this patient population:
  - Left atrial thrombus
  - Recent (less than 3 weeks) ischemic stroke
  - Hemorrhagic stroke
  - Early (less than 4 days) post-operative period
  - Traumatic accident (less than 4 weeks)
  - Bleeding diathesis
  - Intracranial mass
  - INR over 2.5
  - Active internal bleed
  - Aortic dissection

- Baseline Monitoring
  - INR must be less than 2.5
  - aPTT should be less than 50 seconds
  - TEE evaluation at baseline to evaluate for left atrial appendage thrombus or other contraindication

- Low dose Ultraslow Dosing (NYHA I-II):
  - 25 mg over 25 hours (1 mg/hr)
  - Can be repeated up to 8 times with at least 6 hours between each infusion for a maximum total dose of 200 mg
- Low dose slow Dosing (NYHA III-IV):
  - 25 mg administered over 6 hours (4.2 mg/hr)
  - Can be repeated 24 hours later and up to 6 times for a maximum total dose of 150 mg
  - Patients with partial success (residual thrombus size of ≥10 mm) can receive a low dose ultraslow infusion (25 mg over 25 hours, repeated up to 200 mg)
- Low dose Ultraslow and Slow alteplase monitoring
  - Heparin drip should be withheld and aPTT be less than 50 seconds before initiation
  - Withhold all oral anticoagulants including warfarin before initiating alteplase infusion
  - Heparin drip with goal aPTT 53-71 seconds with no initial bolus can be initiated after completion of the alteplase infusion
  - Withhold heparin and ensure aPTT less than 50 seconds in case of need for repeat alteplase
  - Echocardiogram to be repeated after each alteplase infusion

References

1. Özkan M, Gündüz S, Gürso OM, et al. Ultraslow thrombolytic therapy: A novel strategy in the management of PROsthetic MEchanical valve Thrombosis and the prEdictors of outcomE: The Ultra-slow PROMETEE trial. Am Heart J. 2015 Aug;170(2):409-18.
2. Özkan M, Gündüz S, Biteker M, et al. Comparison of different TEE-guided thrombolytic regimens for prosthetic valve thrombosis: the TROIA trial. JACC Cardiovasc Imaging. 2013 Feb;6(2):206-16.

3. Özkan M, Gündüz S, Güner A, Kalçık M, Gürsoy MO, Uygur B, Keleş N, Kaya H, Kılıçgedik A, Bayam E, Kalkan S, Astarcıoğlu MA, Karakoyun S, Yesin M, İnan D, Fedakar A, Sarıkaya S, Aksüt M, Onan B, Koçoğulları CU. Thrombolysis or Surgery in Patients With Obstructive Mechanical Valve Thrombosis: The Multicenter HATTUSHA Study. J Am Coll Cardiol. 2022 Mar 15;79(10):977-989

Abbreviations

1. CCAD : Cleveland Clinic Abu Dhabi
2. t-PA: alteplase
3. NYHA: New York Heart Association
4. INR: international normalized ratio
5. aPTT: activated partial thromboplastin time
